# Towards a neuro-computational account of prism adaptation

**DOI:** 10.1016/j.neuropsychologia.2017.12.021

**Published:** 2018-07-01

**Authors:** Pierre Petitet, Jill X. O’Reilly, Jacinta O’Shea

**Affiliations:** a Wellcome Centre for Integrative Neuroimaging (WIN), Oxford Centre for Functional MRI of the Brain (FMRIB), Nuffield Department of Clinical Neurosciences (NDCN), University of Oxford, John Radcliffe Hospital, Headington, Oxford, UK; b Donders Institute for Brain, Cognition and Behaviour, Radboud University, Nijmegen, Netherlands; c Department of Experimental Psychology, University of Oxford, 9 South Parks Road, Oxford OX1 3UD, UK

**Keywords:** Prism adaptation, Sensorimotor control, Computational neuroscience

## Abstract

Prism adaptation has a long history as an experimental paradigm used to investigate the functional and neural processes that underlie sensorimotor control. In the neuropsychology literature, prism adaptation behaviour is typically explained by reference to a traditional cognitive psychology framework that distinguishes putative functions, such as 'strategic control' versus 'spatial realignment'. This theoretical framework lacks conceptual clarity, quantitative precision and explanatory power. Here, we advocate for an alternative computational framework that offers several advantages: 1) an algorithmic explanatory account of the computations and operations that drive behaviour; 2) expressed in quantitative mathematical terms; 3) embedded within a principled theoretical framework (Bayesian decision theory, state-space modelling); 4) that offers a means to generate and test quantitative behavioural predictions. This computational framework offers a route towards mechanistic neurocognitive explanations of prism adaptation behaviour. Thus it constitutes a conceptual advance compared to the traditional theoretical framework. In this paper, we illustrate how Bayesian decision theory and state-space models offer principled explanations for a range of behavioural phenomena in the field of prism adaptation (e.g. visual capture, magnitude of visual versus proprioceptive realignment, spontaneous recovery and dynamics of adaptation memory). We argue that this explanatory framework can advance understanding of the functional and neural mechanisms that implement prism adaptation behaviour, by enabling quantitative tests of hypotheses that go beyond merely descriptive mapping claims that ‘brain area X is (somehow) *involved* in psychological process Y’.

## Highlights

• Traditional neuropsychological models of prism adaptation lack precision.
• Computational models improve explanatory and predictive power.
• A range of adaptation phenomena can be explained quantitatively.
• Mathematics offers a bridge between neural mechanisms and behaviour.
• A neuro-computational approach will advance neuropsychology.

## 1 Introduction

Adaptation is a fundamental property of the nervous system that enables organisms to flexibly reconfigure sensorimotor processing to counteract perturbations that cause performance errors (Shadmehr et al., 2010, Franklin and Wolpert, 2011). Consider, for example, the case of a basketball player shooting at various times throughout a game. As the game progresses, so muscles will fatigue, such that the same motor command produces a different outcome from one shoot to another. A lateral wind might also alter the trajectory of the ball and deviate it from the aimed basket. In these two situations, an internal (muscle fatigue) or external (wind) disturbance introduces systematic deviations from the intended action goal. These perturbations require the relationship between a desired action goal and the motor commands that execute it to be reconfigured, to avoid the large systematic errors in performance that would ensue if the nervous system were unable to adapt and correct for the perturbations. Thus, adaptation underwrites the maintenance of successful actions across the lifespan.

In a laboratory context, sensorimotor adaptation has been studied experimentally using a variety of methods (e.g. visuomotor rotation, force-field adaptation, saccade adaptation, Coriolis forces, etc.) (Lackner and Dizio, 1994, Shadmehr and Mussa-Ivaldi, 1994, Mazzoni and Krakauer, 2006, Ethier et al., 2008). Here we focus on a method first developed by von Helmholtz at the end of the nineteenth century, called *prism adaptation* (Von Helmholtz, 1867). In this paradigm, participants wear prism glasses that bend light, and so optically displace the visual field, for example by 10° to the right. When participants perform visuo-motor tasks (e.g. pointing at targets) while wearing the prisms, at first, they make systematic rightward errors (owing to the optical displacement), but participants learn rapidly from the error feedback to correct their movements on subsequent trials and regain normal accuracy (i.e. they adapt). When the prisms are removed post-adaptation, individuals then make errors in the opposite direction, i.e. a leftward "after-effect", which reflects the temporary persistence of some of the compensatory mechanisms engaged during the adaptation. Several features of how prism after-effects generalise or transfer beyond the specifically trained context make it an interesting paradigm to investigate. In healthy controls, prism after-effects tend to generalise at least partially across space (Bedford, 1989, Bedford, 1993, Redding and Wallace, 2006b). This contrasts with visuomotor rotation, for instance, where effects drop off sharply with distance from the trained target location (Krakauer et al., 2000). Pointing during prism exposure is typically aimed at lateral targets under speeded conditions, whereas prism after-effects are often measured at a central (untrained) location, with accuracy emphasised over speed. With this procedure, there is therefore a change in task context between prism exposure and prism after-effect measurement conditions, such that the after-effect measure intrinsically captures elements of generalisation/transfer, at least with respect to task changes (training/test or exposure/after-effect) in reach trajectory, movement speed and target location. Prism after-effects are measurable in at least three different modalities, visual, proprioceptive, and motor, which appear to follow different dynamics (Harris, 1963, Redding and Wallace, 2001, Hatada et al., 2006b, Hatada et al., 2006c, Hatada et al., 2006d). It has been claimed that prism after-effects can transfer to untrained visuospatial tasks (e.g. line bisection task, greyscales task), although these effects in young healthy volunteers appear to occur only with left-shifting (not right-shifting) prisms and tend to be quite small and variable (Colent et al., 2000, Michel et al., 2003, Loftus et al., 2009, Goedert et al., 2010, Martin-Arevalo et al., 2014, Schintu et al., 2014, Schintu et al., 2017, Striemer et al., 2016). A stronger evidence base in patients has shown that the after-effects of prism adaptation can transfer to improve cognitive deficits in visuospatial neglect after right hemisphere brain damage (Rossetti et al., 1998, Frassinetti et al., 2002, Serino et al., 2009, O'Shea et al., 2017). After-effects have been shown to transfer to a broad range of untrained sensory and cognitive domains in neglect, including, for example, postural control, occulo-motor exploration, dichotic listening and mental imagery (for review, see: Jacquin-Courtois et al., 2013). Improved symptomatology after prism adaptation has also been reported in patients with complex regional pain syndrome (Sumitani et al., 2007) and Parkinson's disease (Bultitude et al., 2012). This distinctive generalisation/transfer profile of prism adaptation, by contrast with other adaptation paradigms, suggests that this experimental model of sensorimotor integration warrants special attention.

What features should a satisfying theoretical account of prism adaptation behaviour have? An ideal account would provide: 1) mechanistic explanations, that are 2) biologically plausible, and 3) can generate quantitative behavioural predictions, 4) about the effect of a range of factors, such as experimental task manipulations (e.g. modality, quality and timing of sensory feedback, gradual versus abrupt perturbation onset, etc.), psychological variables (e.g. internal state estimates of limb position, sensory uncertainty, prior knowledge of the perturbation, etc.), and neural state effects (e.g. change in neural excitability in specific brain region owing to lesion or drug or brain stimulation intervention). Here, we outline the current prevailing (descriptive psychological) model of prism adaptation that is predominant in the literature on healthy individuals, patients and animal studies. We also highlight the brain regions implicated in prism adaptation by studies conceived within this framework. Next, we make the case that a computational characterisation of prism adaptation behaviour offers advantages over this traditional functional descriptive approach, and argue the need for an integrated neuro-computational account to further advance understanding within this field.

## 2 Prism adaptation procedures

Since the primary focus of this paper is on prism adaptation experiments, we will first describe how such studies are typically performed. This will provide the reader with the necessary background to engage with the prism adaptation literature and understand the theoretical discussion developed in the following sections.

### 2.1 Typical experimental paradigm

**Fig. 1:**
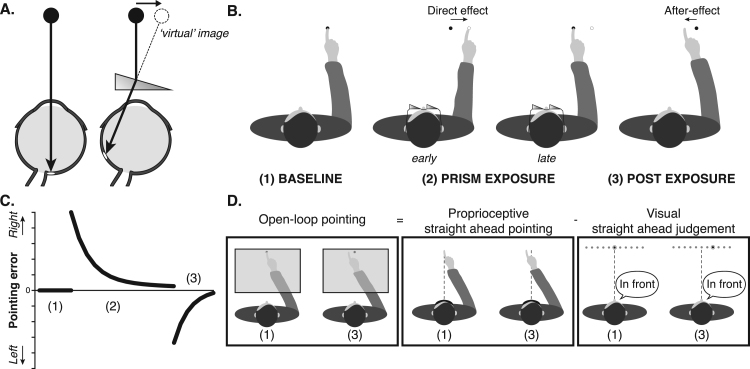
**Prism adaptation (A)** By bending light, prism lenses displace the visual field in a direction determined by the prism structure. Here for example, light is displaced laterally, by 10° to the right. Hence, a central dot when viewed through this prism is (mis)perceived to be located 10° to the right of its true position. **(B) Typical prism adaptation experimental paradigm.** Participants’ pointing accuracy is tested first at baseline (1), prior to prism exposure. Figure illustrates closed-loop pointing at baseline, i.e. participant is required to make fast and accurate pointing movements to a visual target and receives visual feedback of the reach trajectory and endpoint. During prism exposure (2), the goggles shown in A) are worn. Owing to the optical shift, the 'direct effect' is that the participant makes rightward pointing errors initially (early phase), but learns gradually from trial-by-trial error feedback to correct these errors and re-gain baseline pointing accuracy (late phase). Consequent leftward prism after-effects (errors) are measurable post-adaptation once the glasses have been removed (3). **(C) Canonical pattern of performance errors during prism adaptation**. Plot shows reach endpoint error (y-axis) as a function of trial number (x-axis) during closed-loop pointing (i.e. with visual feedback). Note baseline accuracy (i.e. mean error centred on zero) (1), followed by rightward errors (in the direction of the prismatic shift) that decrease gradually across prism exposure trials (2), followed by leftward errors (in the direction opposite the prismatic shift) after removal of the prism goggles (3). **(D) Three tests commonly used in the prism adaptation literature to quantify prism after-effects**. During *open-loop pointing* participants point at visual targets, which are viewed transiently, and visual feedback of the reach trajectory and the reach endpoint is deprived. This prevents (further) learning from endpoint error (which would over-turn the after-effect). *Open-loop pointing* measures of after-effect are deviated in the direction opposite the prismatic shift (i.e. here leftward). During *proprioceptive straight ahead pointing* blindfolded participants are asked to point in the direction they perceive as being straight ahead of their nose. This is thought to capture the proprioceptive component of adaptation. This after-effect measure is also deviated in the direction opposite to the prismatic shift (i.e. leftward). During *visual straight ahead judgement* participants must indicate when a moving light is perceived as being straight ahead of their nose. This is thought to capture the visual component of adaptation. After-effects with this measure are deviated in the same direction as the prismatic shift (i.e. rightward). The sum of visual and proprioceptive after-effects immediately after prism exposure has been shown to equal the magnitude of after-effect quantified by open-loop pointing, which is therefore known as the *total visuomotor shift* (Hay and Pick, 1966, Templeton et al., 1974, Redding and Wallace, 1988, Redding and Wallace, 1996, Hatada et al., 2006a).

Prism adaptation experiments usually include at least three phases: 1) pre-adaptation: baseline measure of individuals’ visuomotor task performance; 2) prism exposure: visuomotor tasks are performed during exposure to the prismatic shift. This optical shift causes performance errors – individuals learn from error feedback to correct their errors in order to compensate gradually for the optical shift (i.e. they adapt); 3) post-adaptation: baseline tests are repeated, and changes in performance (post - pre) provide a measure of *after-effects* (Kornheiser, 1976) (Fig. 1).

In the studies we will consider, participants are typically asked to make pointing movements during each of the three phases. Visual feedback of individuals’ trial-by-trial reach endpoints can either be provided (closed-loop pointing, CLP) or deprived (open-loop pointing*, OLP*). During prism exposure (phase 2), endpoint errors are deviated initially in the direction of the optical displacement on both of these types of pointing. Performance change relative to baseline (during *minus* pre) during this initial phase is often described as the *direct effect* of prisms. If visual feedback of endpoint errors is provided during prism exposure (i.e. closed-loop pointing), individuals tend to reduce these errors progressively to regain baseline accuracy (i.e. they adapt) (Fig. 1B, C).

Prism after-effects are measured by asking participants to point again after removal of the prisms. If sufficient practice has occurred during prism exposure, performance will now be deviated in the direction opposite to the prismatic shift. If visual feedback of these (now leftward) reach endpoints is provided (i.e. closed-loop pointing), performance errors will once again be corrected rapidly (now in the opposite direction) to re-gain baseline accuracy (Fig. 1C). This de-adaptation (or washout) can be limited by depriving visual feedback (i.e. using open-loop pointing) (Fig. 1D). Prism after-effects measured using open-loop pointing can persist even after closed-loop performance has returned to baseline levels (Inoue et al., 2015).

Two other tests are commonly contrasted across the baseline and post-exposure phases, to measure modality-specific prism after-effects: visual straight-ahead judgement and proprioceptive straight-ahead pointing (Redding and Wallace, 1988, Redding and Wallace, 1993, Redding and Wallace, 2001, Hatada et al., 2006a, Hatada et al., 2006d). The idea is to measure the different components hypothesised to underlie prism adaptation: visual, proprioceptive and motor changes (Harris, 1963). The visual straight ahead judgement requires participants to report verbally when a visual stimulus moving laterally across their visual field is perceived as being straight ahead of their nose (Fig. 1D). This measure is thought to rely on eye-head coordination, and any post-exposure change in accuracy is usually interpreted as a visual after-effect (Hatada et al., 2006a). Proprioceptive straight ahead pointing requires blindfolded participants to point to the position in space they perceive to be straight ahead of their nose. It is thought to provide a measure of head-hand coordination, and post-exposure shifts in this measure are interpreted as a proprioceptive after-effect (Harris, 1963, Templeton et al., 1974). Several authors have reported that immediately after prism adaptation, the sum of the visual and proprioceptive after-effects, measured by visual straight ahead judgement and proprioceptive straight ahead pointing, respectively, equals the magnitude of after-effects assayed using open-loop pointing (Hay and Pick, 1966, Templeton et al., 1974, Redding and Wallace, 1988, Redding and Wallace, 1996, Hatada et al., 2006a). This finding is relatively intuitive given that both vision of target location at onset and proprioceptive and motor feedback during the reach trajectory contribute to motor performance during open-loop pointing. Thus, open-loop pointing quantifies the combined contribution of both factors influencing hand-eye coordination (i.e. the total visuomotor shift), while visual straight ahead judgement and proprioceptive straight ahead pointing each measure individual components (eye-head and head-hand respectively). Studies vary in whether they measure just the total visuomotor shift, or the visual or proprioceptive after-effects, or investigate the relationship between all three.

### 2.2 Important factors to consider

Several experimental factors can strongly influence the modality, magnitude and persistence of both the direct- and after- effects of prism adaptation. The way the prismatic shift is introduced (gradually versus abruptly), the visibility of the starting position of the limb, the availability of visual feedback during the movement trajectory versus only at the reach endpoints (i.e. concurrent versus terminal exposure), the duration of prism exposure, the movement speed, the target location, or the limb used - all have been shown to influence behavioural performance (Hamilton, 1964, Bedford, 1989, Redding and Wallace, 1996, Redding and Wallace, 2006a, Kitazawa et al., 1997, Michel et al., 2007, Inoue et al., 2015). Additionally, brain lesions can affect the way individuals adapt to prisms and express after-effects (Bossom, 1965, Welch and Goldstein, 1972, Weiner et al., 1983).

In the following section, we will outline the descriptive theoretical framework typically used within the neuropsychology literature to explain the effects of the various factors listed above. Subsequently, we will argue for the advantages of a computational framework in place of this descriptive account. Key benefits of this formal model framework are that it offers: 1) principled mechanistic explanations of behaviour, 2) which specify the computations that give rise to behaviour, 3) in precise mathematical terms, 4) that enable quantitative tests of behavioural predictions, 5) and characterise information processing in terms (mathematical functions) that could plausibly be implemented by neural circuits (unlike the traditional cognitive psychology descriptive account). We argue that this explanatory framework offers a significant conceptual advance, which promises to accelerate progress in understanding the causal bases of prism adaptation behaviour, in terms of the algorithms that drive it, the neural circuits that implement it, and how these interact.

## 3 The traditional dual-process framework: strategic control versus spatial realignment

### 3.1 Theoretical framework

For the past forty years, the large majority of studies investigating the neural mechanisms underlying prism adaptation have interpreted their results within a theoretical framework that distinguishes two learning processes that contribute differentially to the direct effects (i.e. error correction) and after-effects of prisms. This framework posits that prism adaptation recruits two distinct functional mechanisms: a rapid process of error reduction that reflects strategic adjustments in motor control, and a slower process, so-called ‘true’ sensorimotor adaptation, thought to reflect the spatial realignment of motor, proprioceptive and visual coordinate reference frames (for review, see: Redding et al., 2005; Redding and Wallace, 2006b). The name given to these two processes has varied slightly across studies and over time, but the core idea of a distinction between a fast strategic component and a slower ‘true’ sensorimotor realignment has remained consistent. Here we will adopt Redding and Wallace's (Redding et al., 2005, Redding and Wallace, 2006b) latest terminology to describe this dual-process theoretical framework and review the evidence for functional and anatomical dissociations associated with these two processes.

**Fig. 2:**
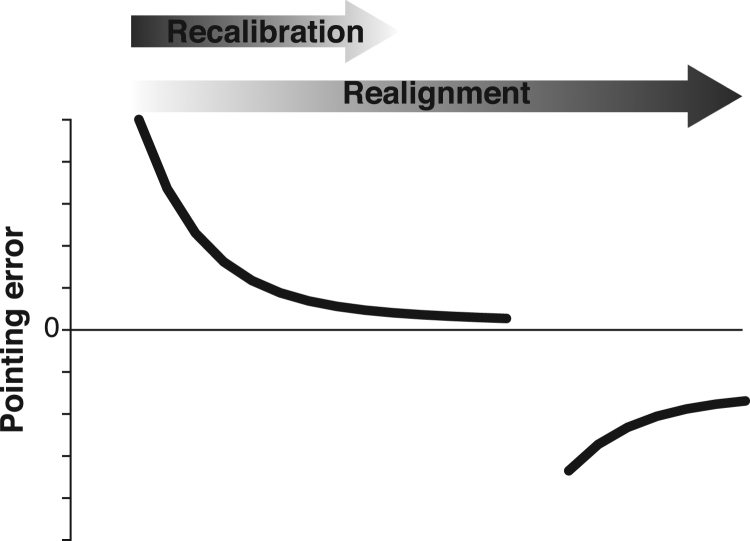
**The traditional dual-process framework.** The traditional theoretical framework posits that error correction during prism adaptation relies on two processes. *Strategic control* refers to the calibration of individuals’ task workspace around the task relevant objects. It is described as a rapid process that intervenes early during prism exposure but contributes poorly to prism after-effects. Conversely, *spatial realignment* is described as developing more slowly during prism exposure and is thought to be responsible for the prism after-effects. The term describes a process of bringing the different sensorimotor coordinate frames (visual-motor, proprioceptive-motor) into alignment with each other.

Within this framework, the constituent processes driving prism adaptation are described as follows. *Strategic control* is a set of processes that guide everyday adaptive motor behaviours. For example, to reach for a cup, depending on the sensory information available, it is necessary to first select the appropriate reference frame to code the target location (e.g. visual-motor, proprioceptive-motor) and guide the appropriate reach-to-grasp command. The process of setting the reference frame in relation to the desired action has been called ‘calibration’ (Redding and Wallace, 2002, Redding et al., 2005). Strategic control also requires selecting the region of extrapersonal space most relevant to the on-going task (e.g. the shelf where the cup is located), and is therefore described as being closely related to spatial attention. If a reach-to-grasp action is unsuccessful, the action may have to be ‘recalibrated’ by various means. For example, (while wearing prisms) one may direct his/her reaching movement more towards the side of the cup, so as to reduce the previous motor error. This mix of partly automatic, partly conscious processes is thought to contribute predominantly to the rapid error correction that occurs during the initial phase of prism exposure, but to contribute poorly to prism after-effects (Weiner et al., 1983, Pisella et al., 2004, Redding et al., 2005, Redding and Wallace, 2006b) (Fig. 2).

It is important to note that, within this framework, strategic motor control adjustments do not require the linkage between different coordinate frames to be reconfigured. Instead, motor performance can be improved simply by changing the motor command issued to reach the target. An analogy commonly used to illustrate this idea is to consider a rifleman with misaligned telescopic sight. Suppose that the scope of the rifle is misaligned with the barrel, so that the marksman misses the target systematically by 10° to the right. Simply re-aiming 10° to the left of the target would allow the marksman to hit his target without having to realign the scope with the barrel. Strategic control refers to this process of quickly recalibrating the reference frame to meet the task objectives. Successful movements driven by such a process should therefore be specific to the trained task context and should not generalise to other contexts (e.g. other movements, other spatial locations, etc.).

By contrast, *spatial realignment* refers to the process of adjusting for constant differences in spatial coordinates between multiple sensorimotor coordinate systems. In reference to the previous analogy, this would be equivalent to realigning the barrel with the scope of the rifle. The traditional theoretical framework often posits that accurate pointing movements require the correct alignment of two reference frames: an eye-head visual-motor system (measured with the visual straight ahead test) and a head-hand proprioceptive-motor system (measured with straight ahead pointing) (Redding and Wallace, 2002, Redding et al., 2005). Because the prismatic shift displaces the visual-motor reference frame only, compensatory shifts in the visual-motor and/or proprioceptive-motor systems are required in order to re-align the two reference frames and regain correct eye-hand coordination during prism exposure (Templeton et al., 1974). The temporary carry-over of these adaptive shifts after removal of prisms is thought to be predominantly responsible for the after-effects of prism adaptation.

### 3.2 Behavioural predictions

In summary, the core theses of the traditional dual-process framework are that: 1) strategic recalibration is a cognitively demanding process, that drives error correction early during prism exposure, but contributes little to prism after-effects; 2) spatial realignment is an automatic process, that develops more gradually, and is mainly responsible for prism after-effects; 3) these two processes operate relatively independently from one another (Fig. 2). Within this theoretical framework, closed-loop performance during prism exposure reflects both the strategic control and spatial realignment components, whereas prism after-effects (measured via open-loop pointing, visual straight ahead judgement or proprioceptive straight ahead pointing) provide a measure of spatial realignment. Impaired strategic control would therefore affect error correction during prism exposure, but would not directly affect prism after-effects. By contrast, impaired spatial realignment would affect both direct- and after- effects of prisms.

In the following two 3.3, 3.4, we will review some of the main empirical evidence that supports this theoretical model (for in depth review, see: Redding and Wallace, 2002; Redding et al., 2005; Redding and Wallace, 2006b) and summarise attempts to localise the neural correlates of these two proposed processes.

### 3.3 Strategic control

Within the traditional framework, strategic control refers to the process of quickly recalibrating the reference frame to (re)code the location of the target. It is thought to contribute mainly to the early phase of prism exposure.

Consistent with this view, several studies have reported behavioural markers of a learning component engaged during the early phase of prism exposure, but saturating quickly, and contributing poorly to the after-effect. For example, in healthy individuals, during the initial phase of prism exposure, when errors are large, the time gap between target foveation and onset of the pointing movement increases transiently, but returns to normal within about 10 trials, as the endpoint error magnitude is rapidly reduced (Rossetti et al., 1993). A similarly rapid time course was observed in trial-by-trial corrections of the initial acceleration phase of the reach trajectory during the first 10 prism exposure trials (O'Shea et al., 2014). It has also been shown that imposing a cognitive load (mental arithmetic) during prism exposure disrupts participants’ ability to correct pointing errors while wearing prisms (Redding et al., 1992). None of these behavioural phenomena have been shown to relate quantitatively to measures of prism after-effects. Taken together, these results implicate a strategic learning component that contributes to error correction during early prism exposure.

The neural substrates associated with this 'strategic control' component of prism adaptation have been inferred from brain lesions that have impaired error reduction during prism exposure but spared after-effects (Welch and Goldstein, 1972, Weiner et al., 1983, Canavan et al., 1990, Pisella et al., 2004, Newport and Jackson, 2006, Fernandez-Ruiz et al., 2007) (Table 1). Such studies have converged on a crucial involvement of the cerebral cortex in strategic control (Welch and Goldstein, 1972, Weiner et al., 1983, Canavan et al., 1990), more specifically, the posterior parietal cortex (Pisella et al., 2004, Newport and Jackson, 2006). The functional specificity implied by this behavioural pattern is debatable given the variety of neurological impairments that can lead to this pattern of performance. For example, temporal lesions (Canavan et al., 1990), spinocerebellar ataxia type 2 (Fernandez-Ruiz et al., 2007), and spatial neglect (Aimola et al., 2012) have all been associated with impaired error reduction but intact prism after-effects.

**Table 1:** **Overview of the lesion studies of prism adaptation.** This table includes the main lesion studies of prism adaptation in humans. It is not meant to be exhaustive. R = right; L = left; PMC = Pre-motor Cortex; PPC = Posterior Parietal Cortex; CLP = Closed-Loop Pointing; OLP = Open-Loop Pointing; SAP = Straight Ahead Pointing; VSA = Visual Straight Ahead judgement.

**Study**	**Study type**	**n**	**Etiology**	**Shift**	**Hand**	**Error reduction**	**After-effect**	**Impaired strategic control**	**Impaired spatial realignment**
***Cerebellar lesions***									
Weiner et al., 1983	Patient group	7	Degenerative (n = 5), infarct (n = 1), drug induced (n = 1)	R	R/L	Impaired	Reduced on CLP		X
Baizer et al., 1999	Animal lesion	1	Aspiration lesion: left posterior cerebellum, including caudal parts	L	L/R	Impaired	Impaired on CLP		X
		4	As above but not including the caudal parts	L	L/R	Normal	Normal on CLP		
Pisella et al., 2005	Case study	1	Left anterior cerebellar stroke	L	R	Not reported	No aftereffect on OLP		X
				L	L	Not reported	No aftereffects on OLP		
				R	R	Not reported	Aftereffects on OLP		
				R	L	Not reported	Aftereffects on OLP		
Fernandez-Ruiz et al., 2007	Patients group	43	Spinocerebellar ataxia type 2 (SCA2)	R	R	Impaired	Normal on OLP	X	
Calzolari et al., 2015	Case study	1	Left lateral cerebellar stroke	R	R	Normal	Normal on OLP		(X)
							Normal on VSA		
			+ bilateral occipital lesion				Aberrant rightward after-effect on SAP		
Hanajima et al., 2015	Group study	13	Cerebellar cortical atrophy	R/L	R	Impaired	Reduced on OLP		X
Hashimoto et al., 2015	Group study	77	Degenerative cerebellar diseases	R	R	Impaired	Reduced on CLP		X
***Parietal lesions***									
Pisella et al., 2004	Case study	1	Bilateral PPC stroke	L	R	Impaired	Normal on OLP	X	
Newport and Jackson, 2006	Case study	1	Bilateral PPC atrophy, predominantly in the left hemisphere	R	R	Abolished	After-effect on OLP	X	
				R	L	Impaired	No after-effect on OLP	X	(X)
Newport et al., 2006	Case study			R	R	Abolished	No after-effect on OLP	X	(X)
				R	L	Abolished	No after-effect on OLP	X	(X)
***Others***									
Welch and Goldstein, 1972	Patient group	18	Various brain lesions	L	R	Impaired	Reduced on OLP	X	
							Normal on SAP		
Weiner et al., 1983	Patient group	15	Left hemisphere lesion	R	R/L	Normal	Normal on CLP		
		14	Right hemisphere lesion	R	R/L	Impaired	Normal on CLP	X	
		10	Parkinson's disease	R	R/L	Slightly impaired	Normal on CLP	(X)	
Canavan et al., 1990	Patient group	9	Frontal lesions	?	R	Impaired	Normal on CLP	X	
		9	Left temporal lobectomy	?	R	Normal	Normal on CLP		
		10	Right temporal lobectomy	?	R	Impaired	Normal on CLP	X	
		19	Early Parkinson's disease	?	R	Impaired	Normal on CLP	X	
Stern et al., 1988	Patient group	14	Parkinson's disease	L	R	Normal	Reduced on CLP		X
Fernandez‐Ruiz et al., 2003	Patient group	35	Parkinson's disease	R	R	Normal	Reduced on CLP		X
Aimola et al., 2012	Patient group	11	Right hemisphere lesion with spatial neglect	R	R	Impaired	Normal on OLP	X	

In the healthy brain, several groups have used neuroimaging techniques to try and identify brain regions involved in strategic control during prism adaptation. Typically, these studies have contrasted the amplitude of neural activity in the ‘early’ versus ‘late’ phases of prism exposure and interpreted areas activated by this contrast (i.e. early > late) as neural correlates of the strategic control component of prism adaptation (Table 2). Consistent with lesion studies, activity in the posterior parietal cortex has been reliably reported by whole brain neuroimaging studies, but other brain regions such as primary motor cortex, anterior cingulate cortex or cerebellum have also been found to be preferentially activated during the early versus late phase of prism exposure (Clower et al., 1996, Danckert et al., 2008, Luauté et al., 2009, Kuper et al., 2014) (Table 2). Aside from functional localisation in this way, these neuroimaging studies have said little about *how* these identified brain regions are thought to implement the process of strategic control.

**Table 2:** **Overview of the neuroimaging studies of prism adaptation.** * Study including the cerebellum and dentate nucleus only. R = right; L = left; IPS = Inferior Parietal Sulcus; M1 = Primary Motor Cortex; ACC = Anterior Cingulate Cortex; SPL = Superior Parietal Lobule; cDN = caudate Dentate Nucleus; STS/STG = Superior Temporal Sulcus/ Superior Temporal Gyrus.

**Study**	**Prismatic shift**	**Pointing hand**	**Strategic control**						**Spatial realignment**					
			**Analysis**	**Area**	**Side**	**MNI coordinates**			**Analysis**	**Area**	**Side**	**MNI coordinates**		
						***x***	***y***	***z***				***x***	***y***	***z***
Clower et al., 1996	Left/Right	Right	*Prism adaptation task > Error correction task*	IPS	L	-51	-49	44						
Danckert et al., 2008	Right	Right	*Early prism pointing trials (trials 1-3) > Later prism pointing trials (trials 7-10)*	M1	L	-36	-16	44						
				ACC	R	2	-1	49						
				Anterior IPS	L	-39	-40	51						
				Medial cerebellum	R	1	-62	-4						
Luauté et al., 2009	Left	Right	*Early prism block (run 2) > Late prism block (run 3)*	Anterior Cerebellum	R	24	-38	-32	*Late prism block (run 3) > Early prism block (run 2)*	STS/STG	R	58	-12	-12
												70	-20	4
				IPS/SPL	R	40	-54	66		STS/STG	L	-60	-26	4
				IPS/IPL	L	-30	-68	40						
Chapman et al., 2010	Right	(Right)							*Late prism blocks (run 3-4) > Early pointing blocks (run 1-2)*	Posterior cerebellum	R	16	-58	-50
										Angular gyrus	R	36	-78	30
										Anterior IPL	R	42	-50	46
Kuper et al., 2014*	Right	Right	*Early prism block > Late prism block*	Posterior cerebellum (Lobule VIII)	R	31	-57	-52	*Late prism block*	Posterior cerebellum (Lobule VI)	R	3	-60	-19
				Posterior cerebellum (Lobule IX)	R	6	-62	-42						
				cDN	R	13	-67	-32						
						18	-66	-36						

### 3.4 Spatial realignment

Within the traditional dual process framework, spatial realignment refers to the process of shifting the visual-motor and/or proprioceptive-motor systems in order to re-align these two coordinate frames and regain accurate hand-eye coordination. This process is believed to develop slowly during prism exposure, and to be responsible almost entirely for the prism after-effects (Redding and Wallace, 2002, Redding and Wallace, 2006b, Redding et al., 2005).

One study in healthy individuals reported a putative kinematic signature of sensorimotor realignment: gradual correction of the terminal (deceleration) phase of the pointing trajectory during prism exposure. This corrective process unfolded slowly during prism exposure and the magnitude of correction of this kinematic error correlated with the magnitude of prism after-effects (O'Shea et al., 2014). Endpoint error appears not to be necessary for spatial realignment to occur, as after-effects can be observed in the absence of measurable reach endpoint errors if the prismatic shift is introduced gradually (Howard and Freedman, 1968, Dewar, 1971, Michel et al., 2007, Hanajima et al., 2015). Instead, it has been suggested that the discordance between the expected (feedforward predicted) and observed (feedback measured) position of the hand is the learning signal for spatial realignment (Redding and Wallace, 2006b). Support for this claim comes mainly from the finding that the magnitude of visual and proprioceptive after-effects (measured by the visual straight ahead judgement and proprioceptive straight ahead pointing, respectively) depends upon the sensory information available in-flight during the reach trajectory when individuals are wearing prisms (Redding and Wallace, 1996, Redding and Wallace, 2001). If both proprioceptive and visual feedback is available, proprioceptive after-effects tend to be greater than visual after-effects. The opposite is true (i.e. greater visual than proprioceptive after-effects) if only proprioceptive feedback is available (Redding and Wallace, 1996, Redding and Wallace, 2001). This suggests that the modality in which the discrepancy between the predicted and observed hand location is sensed determines which coordinate reference frame is preferentially re-aligned.

Several studies have reported evidence of impaired spatial realignment following cerebellar lesions in humans (Weiner et al., 1983, Martin et al., 1996, Pisella et al., 2005, Calzolari et al., 2015, Hanajima et al., 2015) (Table 1) and non-human primates (Baizer et al., 1999). The typical behavioural pattern is reduced error correction during prism exposure, combined with decreased or absent prism after-effects. The contribution of the precise cerebellar sub-regions is still unclear, as evidence of impaired spatial realignment has been found after anterior (Pisella et al., 2005, Calzolari et al., 2015) and posterior (Martin et al., 1996, Baizer et al., 1999) lesions to cerebellum. To our knowledge, only three neuroimaging studies have investigated the pattern of functional brain activity associated with spatial realignment (Luauté et al., 2009, Chapman et al., 2010, Kuper et al., 2014). They did so by investigating brain regions that were more active during the later stage of prism exposure compared to the early stage (late > early). Posterior cerebellar activity was reported in two of these studies (Chapman et al., 2010, Kuper et al., 2014), but other regions such as the superior temporal gyrus and angular gyrus were also activated (Luauté et al., 2009, Chapman et al., 2010) (Table 2).

### 3.5 Is this theoretical framework satisfying?

The traditional dual-process theoretical framework offers an account of various behavioural dissociations observed in healthy individuals and neurological patients (for review, see: Redding and Wallace, 2002; Redding et al., 2005; Redding and Wallace, 2006b). The main functional insight provided by this framework has been to distinguish two psychological processes (strategic control, spatial realignment) that combine to explain behaviour, but seem to operate with a certain degree of independence from one another. However, studies conceived within this theoretical framework do not offer a mechanistic explanation of how prism adaptation behaviour arises, and attempts to localise the neural circuits underlying these two processes have yielded heterogeneous results (Table 1). In addition, the information processing operations executed by the identified neural components remain largely unknown.

The traditional dual-process theory suffers from a pervasive problem in cognitive neuroscience – how to bridge the conceptual gap between cognitive psychological level descriptions of behaviour and biologically plausible descriptions of neural circuit functioning? Computational models offer a potential bridge, as they provide a common currency (algorithms, mathematical functions) in which to describe both information processing and neural circuit mechanisms that could implement such functions. We contend that, in order to advance cognitive neuroscience explanation of the causal brain-behaviour dynamics that underwrite prism adaptation, the traditional dual-process framework needs to be replaced with a re-conceptualisation at the algorithmic level. By ‘algorithmic’, we mean a level of description that sets out clearly the (mathematical) rules and operations (functions) required to execute prism adaptation behaviour. It is obvious that, at the cellular and neural circuit level, cognitive concepts like ‘strategic control' or ‘spatial realignment’ have no explanatory value, since brain circuits are computing information, and the explanatory task is to provide an account of how these computations implement psychological functions. Explanatory progress therefore requires a conceptual advance: a theoretical framework that decomposes prism adaptation behaviour into the underlying algorithms required to implement it (for a related recent argument, see: Krakauer et al., 2017). Re-conceptualising prism adaptation in terms of its constituent algorithms offers objective mathematical description, as opposed to qualitative description offered by the traditional dual-process psychological framework. This greater precision helps avoid confusion related to terminology. It also allows for quantitative experimental predictions. We will return to these points in the next section. More generally, recasting any behaviour or cognitive process in algorithmic terms helps advance the field towards a re-defined taxonomy of cognitive processes, one grounded in the recognition that the same computations (and brain circuits) might contribute to diverse behavioural phenomena depending on the constraints of the task. This offers a way to move beyond accounts of brain activations in terms of 'area X is *involved* in cognitive function Y (e.g. attention)' to 'computation Y is *implemented* within circuit X and engaged during tasks a, b, c'. This distinct conceptual approach could, for example, help to explain why prism after-effects transfer from pointing tasks to cognitive domains in neglect patients (Rossetti et al., 1998, Sumitani et al., 2007).

In the following section, we propose an algorithmic decomposition of the computations required for prism adaptation, by leveraging insights from the field of computational neuroscience applied to sensorimotor control. We argue that this decomposition, which is not incompatible with the traditional psychological approach, offers a more useful theoretical framework, in terms of quantitative precision, explanation, and plausible neural implementation.

## 4 Computational principles of sensorimotor control

### 4.1 The temporal dynamics of adaptation explained by multiple timescale models

**Fig. 3:**
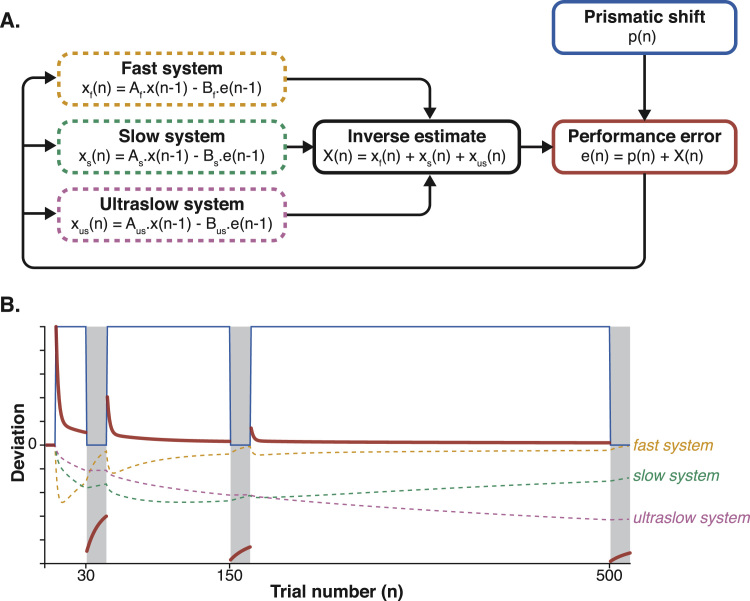
**The three timescales state-space model for prism adaptation. (A)** Each of the fast, slow and ultraslow systems are described by a pair of free parameters: a retention factor A, that describes the amount of decay occurring between trials (0 < A _f_ < A_s_ < A_us_ < 1), and a learning rate B, that describes the fraction of performance error being incorporated into that system's state on each trial (0 < B_us_ < B_s_ < B_f_ < 1). The sum of the states of the three systems produces an inverse estimate of the perturbation on a trial-by-trial basis, which can be used to correct motor output. On any trial, performance error therefore corresponds to the sum of the prismatic shift and the state of the three systems. We have chosen here to illustrate state-space models with a three timescales model because of its relevance for prism adaptation (Inoue et al., 2015), but in principle any number of systems could be posited. **(B)** Simulation of prism adaptation in the three timescales model, based on Inoue et al. (2015) experiment. When the prismatic shift (in blue) is introduced, the three systems (dotted yellow, green and purple lines) learn at three different rates (set by their respective learning rates B). The sum of the states of the three systems is added to the magnitude of the prismatic shift to reduce performance error (in red) during prism exposure. If the after-effects are probed after 30 exposure trials (learning rate is set to 0 for all three systems during after-effect trials, i.e. open-loop pointing. shadded grey), the memory trace decays rapidly, because it is largely dominated by the fast system, which has the lowest retention factor. However, if prism exposure continues, the contribution of the fast system gradually decreases. As a result, after-effects are more stable after 150 trials. Finally, after extended prism exposure of 500 trials, the ultraslow system accounts for most of the net adaptation (i.e. the purple line is the most negative one of all three). Because of that system's high retention factor, the after-effects are then very stable. (For interpretation of the references to color in this figure legend, the reader is referred to the web version of this article.)

State-space modelling has provided insights into the hidden internal state learning and forgetting dynamics that contribute to overt behaviour during sensorimotor adaptation (Smith et al., 2006). Trial-by-trial error correction has been explained as the output of multiple internal adaptive systems that learn and forget on different timescales. The key idea is that these systems compete to learn from performance error and that the sum of their states provides an inverse estimate of the perturbation that is used to correct motor performance (Fig. 3A). A commonly used simplified model posits that the multitude of possible timescales can be approximated by a *fast system*, which learns and forgets rapidly, and a *slow system*, which learns and forgets more slowly (Smith et al., 2006). The model posits that early during adaptation, error correction is mainly dominated by the fast system, which saturates quickly before decaying back to baseline. By contrast, the slow system develops more gradually over extended practice and accounts for most of the net adaptation at later stages of exposure to the perturbation. This ‘two-state’ framework is able to reproduce and explain a range of behavioural phenomena in adaptation, including apparently counterintuitive effects such as spontaneous recovery - the re-appearance of after-effects after a brief period of washout (Smith et al., 2006, Zarahn et al., 2008, Lee and Schweighofer, 2009). The model offers an efficient way to extract (hidden, internal state-driven) temporal dynamics of learning processes that drive adaptation and to generate and test precise quantitative predictions about the relative contributions of these fast versus slow systems to behaviour. For example, it has been shown that the level of adaptation reached by the slow system during force-field adaptation, rather than the overall performance improvement, predicts the amount of long-term retention (Joiner and Smith, 2008).

Because they are not restricted to two learning systems (Kording et al., 2007, Inoue et al., 2015, Kim et al., 2015), multiple timescale models can also be used to ask what number of processes best explains adaptation behaviour. For example, a recent prism adaptation study asked how many systems were needed to explain the immediate and long-term retention dynamics of prism after-effects (Inoue et al., 2015). During prism exposure, error-dependent learning (closed-loop trial accuracy) asymptotes quickly after the initial error correction phase, i.e. once pointing accuracy has been restored to near-baseline levels, it remains at the same level for the rest of the exposure period. However, even once CLP behaviour has plateaued, the subsequent after-effect dynamics (on open-loop trials) continue to change, depending on the duration of the preceding prism exposure. Typically, the longer the prism exposure, the more stable the subsequent after-effects (i.e. less decay), reflecting consolidation of the acquired adaptation. To account for this phenomenon, Inoue and colleagues proposed a third ‘ultraslow’ system, required to explain behaviour only when prism exposure was prolonged (500 trials) (Fig. 3B). Using a Bayesian model with an even larger number of states to explain saccadic adaptation, Kording et al. (2007) argued that these internal states reflect the nervous system's current estimate of the perturbation timescale, which shifts towards slower, longer timescale estimates as a function of increasing exposure duration (Kording et al., 2007). In other words, optimal adaptation (learning and forgetting rates) reflects the solution of a temporal credit assignment problem: what is the most likely timescale of the perturbation that causes the observed performance error? A Bayesian solution treats longer exposure duration as greater evidence of a long-lasting perturbation. Hence, at any time point, optimal behaviour is defined as the motor output most consistent with the evidence of the estimated perturbation (magnitude and timescale). This theoretical framework has been shown to reproduce and explain a variety of saccade adaptation behaviour in both monkey and human (Kording et al., 2007). In summary, there may be a continuum of learning processes, operating over multiple timescales, whose contribution to behaviour varies with factors such as the source, duration, and volatility of the perturbation.

Following similar reasoning, a recent study combined state-space modelling and functional magnetic resonance imaging (fMRI) to test for neural signatures associated with a range of possible internal state learning and forgetting dynamics during adaptation to a novel visuomotor rotation (Kim et al., 2015). The rationale was to use state-space modelling to extract hidden internal states and identify brain regions where the blood oxygenation level dependent (BOLD) signal co-varies with these computational dynamics. Using a dimensionality reduction analysis, Kim and colleagues identified four main components (i.e. group of multiple timescales), each associated with different neural networks. Notably, all these networks included either a parietal and/or a cerebellar component, suggesting that the functional activation timecourses of brain regions known to be associated with prism adaptation (see Section 3) are compatible with the state-space modelling framework. In a different field, recent advances in biophysical models of neural networks also support the idea that different brain networks process information at different timescales (Fusi et al., 2005, Bernacchia et al., 2011, Chaudhuri et al., 2015). This may offer a potential path to bridge biologically plausible models of neural networks with computational descriptions of learning systems and span explanatory levels from neurons to behaviour.

In summary, computational models including multiple time-invariant adaptive systems offer a good quantitative description of motor behaviour during sensorimotor adaptation. The main theoretical limitation of this approach is its agnosticism regarding the information content that is being learnt (and forgotten) by these multiple systems during adaptation. Prism adaptation induces a visuo-proprioceptive conflict that requires the nervous system to modify sensory (visual, proprioceptive) and motor information processing to regain normal behavioural accuracy. What is the relationship between these functional components (visual, proprioceptive and motor systems) and the learning dynamics extracted by state-space models? The following sections outline a computational framework (Bayesian decision theory) that clarifies the content of information processing in relation to the visual, proprioceptive and motor systems.

### 4.2 Internal models for sensorimotor control

Most theoretical models of sensorimotor control posit that prior to generating a successful action (e.g. pointing to a visual target), the nervous system should produce a coherent, accurate and unbiased estimate of the current state of one's body, the external world, and how they interact, based on all useful sources of information available. Based on this knowledge, an action plan can then be selected, one that is most likely to maximise performance in relation to the current behavioural goal. The selection of the most appropriate action plan is fundamentally a decision process that is tightly coupled to the ability to generate accurate predictions about the consequences of one's actions on the world and/or one's body (Kording and Wolpert, 2006).

Within this framework, sensorimotor control is proposed to rely on internal models of the external world and the mechanics of the body. Inverse motor models transform a desired behavioural goal into an action plan to accomplish it (Wolpert and Kawato, 1998, Kawato, 1999). When applied to the sensory domain, inverse models infer the current state of the body (e.g. position of the hand in space) from sensory input (e.g. proprioceptive feedback). Forward models work in the opposite direction and predict the next state (e.g. next position of the hand) based on an estimate of the current state, a copy of the motor command (efference copy), and some internal representation of the complex causal relationship between the two (for review, see: Miall and Wolpert, 1996; Davidson and Wolpert, 2005; Lalazar and Vaadia, 2008; Shadmehr et al., 2010; Franklin and Wolpert, 2011). In the context of this paper, we will distinguish between a visual and a proprioceptive forward model, each of which generates predictions about the likely next (i.e. expected) visual and proprioceptive state. One advantage of continuously predicting the next state of the body is that this limits the impact of neural transmission delays inherent in relying on actual sensory feedback instead of feedforward predictions (Shadmehr et al., 2010). It also allows the brain to compare continuously the veridicality of its predictions against the actually observed (i.e. sensed) measure of a state (provided by sensory inverse models). Deviations between predicted and observed states (i.e. prediction errors) can be used as a signal to drive updating of internal models (see Section 4.3.3).

### 4.3 Bayesian decision theory

In this section, we outline a theoretical framework that offers a mathematical description of the concepts introduced above (in Section 4.2). Bayesian decision theory is composed of two components, Bayesian statistics and decision theory. Bayesian statistics offers a means to formalise how an ideal observer should combine new information (e.g. observed sensory input) with prior beliefs (e.g. predicted sensory input), and how multiple sources of uncertain information (e.g. multiple sensory modalities, predictions, prior knowledge) should be integrated, in an *optimal* fashion, to generate a more certain combined estimate of the current state. Decision theory describes the process of rationally selecting actions, based on the predictions of internal models, given the current behavioural goal. Bayesian decision theory therefore provides a general framework to formalise optimal state estimation and action selection in a dynamic and uncertain world.

#### 4.3.1 Decision theory and rational movement planning

**Fig. 4:**
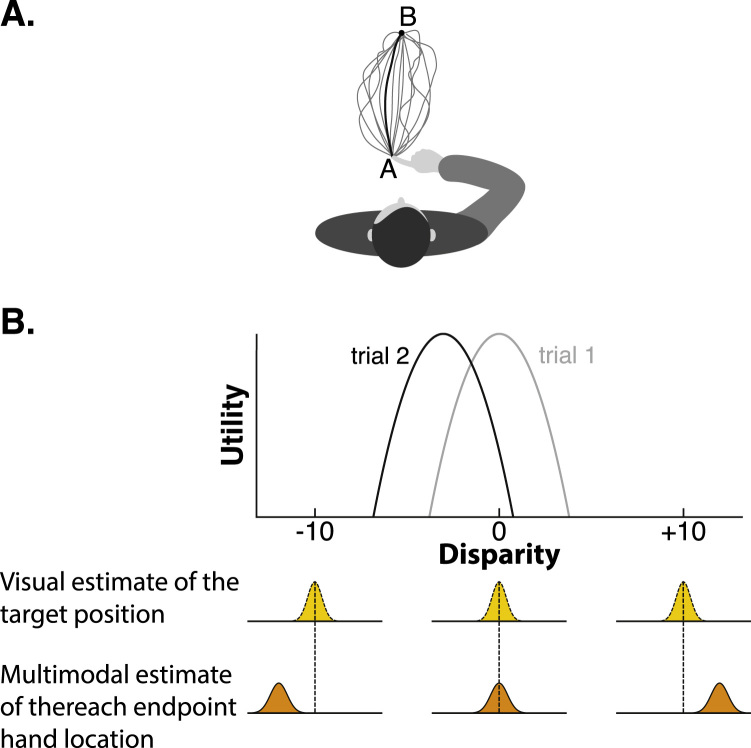
**Decision theory. (A)** Any action can be executed in an almost infinite number of ways. For example, many different movement trajectories would bring one's finger from point A to a point B. Decision theory provides a mathematical framework that describes how a rational decision maker should choose among alternative movement parameters based on their relative level of expected utility. **(B)** Prism adaptation can be conceptualised as a manipulation that affects the computation of expected utility associated with the disparity between the visual estimate of the position of the target and the multimodal estimate of the hand location at the reach endpoint. Before prism onset, the movement plan with highest expected utility is the one that minimises this disparity (i.e. peak utility centred on zero on trial 1). After prism onset however, the visuo-proprioceptive conflict introduced by the prisms induces a performance error: the (experienced) utility of the executed movement plan doesn’t match the predicted utility. This should result in a shift of utility in the direction opposite to the error when planning the pointing movement on trial 2: now, the movement plan with highest expected utility is one that results in a negative disparity between the visual estimate of the target position and the multimodal estimate of the hand location at the reach endpoint (i.e. the multimodal estimate of the hand location is to the left of the visual estimate of the target).

Any given action (e.g. grasping a cup, hitting a ball) can be executed by an almost infinite number of possible movements (Fig. 4). Yet, individuals often move in a very stereotypical way (Morasso, 1981, Harris and Wolpert, 1998, Simmons and Demiris, 2005). Why is this so? Decision theory provides a mathematical framework that formally explains why individuals ‘choose’ to move the way they do. The central concept is that the cost of a potential movement (e.g. energy consumed, fatigue, risk of an injury, etc.) is weighed against the potential reward that is expected from that movement (e.g. sporting success, monetary gain, altruistic feelings, etc.) (Mazzoni et al., 2007, Shadmehr et al., 2016). Mathematically, the concept of a *utility function* (or *cost function*, the negative counterpart) captures the complex relationship that integrates all of these factors, and quantifies the overall desirability of a potential movement choice. Within this decision theoretic framework, the process of selecting one action amongst alternatives is operationalised as the rational choice of whichever movement plan that maximises expected utility (or minimises expected cost) (Kording and Wolpert, 2006, Berniker and Kording, 2011). Mathematically, expected utility is defined as:

(1) $$\mathrm{Expected}\;\mathrm{Utility}\left(\mathrm{movement}\mathrm{plan}\right)=\;p\left(outcome|\;movement\;plan\right).U(\mathrm{outcome})$$

where *p(outcome|movement plan)* is the current estimate of the probability of obtaining an outcome given a particular movement plan (e.g. probability of reaching the target given a certain aiming direction) and *U(outcome)* is the utility associated with this outcome. This mathematical definition becomes intuitive if you consider, for example, a gambling task in which individuals have to choose between alternative options that are associated with varying reward probabilities and reward magnitudes (for example: Hsu et al., 2005; Behrens et al., 2007). In this scenario, the expected utility of each action alternative is the probability of that action yielding a reward, multiplied by the reward magnitude. Choosing the option that maximises expected utility is the definition of choosing rationally. This mathematical framework has been shown to provide a good quantitative fit to human behavioural data in this kind of decision making task (Hsu et al., 2005, Behrens et al., 2007) and has been used to identify functional brain imaging signals that co-vary with the computation of these decision variables (O'Doherty et al., 2003, Daw et al., 2006, Hampton et al., 2006).

When planning a movement, more than one decision needs to be made (Fig. 4). For example, reaching to visual target requires selection of an aiming location (under prism displacement this need not overlap with the estimated target position see Fig. 4B), as well as movement speed and movement trajectory. These different aspects of movement planning are likely to involve different utility functions, and decision theory offers a quantitative framework within which these can be integrated to determine rational movement decisions. Whereas in the gambling task example, the experimenter imposes a utility function, by setting the reward structure of the task, in real-life motor control contexts, it is hypothesised that an agent's rational choice of movements is guided by internal utility functions. Assuming that individuals naturally choose actions that maximise expected utility, then investigating the rules that dictate individuals’ movement properties offers a window onto the internal utility functions that guide action choices. When generating target directed movements, for example, it has been shown that healthy individuals favour the precision rather than smoothness of movement (Harris and Wolpert, 1998), so as to adopt a trajectory that minimises what resembles the terminal squared error (Kording and Wolpert, 2004b). Movement speed seems to be determined by both a speed-accuracy trade-off and an implicit cost assigned to the metabolic energy consumed to produce the movement (Mazzoni et al., 2007, Shadmehr et al., 2016).

Within a decision theoretic framework, factors that change expected utility should result in a change of movement plan. The prism adaptation task can be conceptualised as a manipulation that changes the expected utility of pointing movements. Based on Eq. (1), there are two ways of modifying the expected utility: 1) by changing the utility associated with certain movement outcomes (*U(outcome)),* and/or 2) by changing the probability of obtaining a certain outcome given a movement plan (*p(outcome|movement plan)*). These two could be argued to map on to the traditional dual-process model distinction between strategic control and spatial realignment, respectively.

Strategic control can be re-conceptualised as changing *U(outcome)*, where the outcome is defined as the disparity between the estimate of the hand position at the reach endpoint and the estimate of the target position. Normally, peak utility is when this disparity is zero, i.e. the pointing finger lands on the same location as the (visual) target. However, when an individual corrects strategically for a rightward displacement, by choosing not to aim at the perceived (right-shifted) target location, but instead to aim left of that perceived location, s/he is effectively defining a new utility function with its peak at a non-zero disparity (Fig. 4B). Evidence that movement plans are sensitive to changes in *U(outcome)* has been found in an experiment that imposed new utility functions by modulating the monetary value associated with certain movement outcomes (Trommershäuser et al., 2003). Under these imposed task constraints, healthy individuals were able to rationally select movement endpoints that maximised the potential reward.

Spatial realignment can be conceptualised, within this framework, as minimisation of the prediction error associated with the expected *p(outcome|movement plan)*. That is, the prism manipulation changes the probability that a planned pointing movement (with a given aiming location) will accurately hit the target (*p(hitting the target|aiming location)*). Because of the optical shift, during the early phase of prism exposure the experienced utility of pointing movements does not match predictions (*U(missing the target by 10° to the right) < U(hitting the target)*), i.e. there is a prediction error in terms of utility. We propose that this signal should induce changes in internal models in order to update the computation of *p(outcome|aiming location)*. This would gradually displace the peak of expected utility towards a different movement plan (i.e. more left-oriented) to achieve the desired (unchanged) outcome (i.e. point accurately at the target).

It is worth noticing that this explanatory framework can thus incorporate the key feature of the traditional theoretical framework, i.e. the dissociation between two alternative ways to reduce motor errors during prism exposure (Redding et al., 2005, Redding and Wallace, 2006b) (see Section 3.1). A potential criticism of this proposed decision theory framework is that it merely re-describes the traditional dual-process model and does not add anything new to the understanding of prism adaptation. The answer to this objection is threefold. First, there is value in precise quantitative (mathematical) description of algorithms that explain behaviour, by contrast with qualitative description. Re-specifying behaviour at an algorithmic level also offers a means to generate and test hypotheses about the neural implementation of those computations (O'doherty et al., 2007, Krakauer et al., 2017). Second, the model we propose offers a formal mathematical description of how ‘strategic control' and ‘spatial realignment’ processes interact, which is typically vague or lacking in the traditional framework. Third, this framework incorporates the contribution of both task errors (i.e. breaches of expectancy in terms of the utility of a movement) and reinforcement learning (Sutton and Barto, 1998) in driving behavioural change during adaptation (Huberdeau et al., 2015, Krakauer et al., 2017). A recent adaptation experiment using visuomotor rotation demonstrated this interaction, by showing that rates of adaptation and retention were differentially modulated by monetary rewards and punishments associated with movement outcomes (Galea et al., 2015). Changes in expected utility differentially affected the rate of error correction and the magnitude of subsequent after-effects. This computational framework may also be useful in understanding prism adaptation impairments observed in certain neuropsychological disorders. For example, it has been hypothesised that cognitive impairments observed in patients with Parkinson's disease arise owing to altered utility functions because of depleted dopamine in the basal ganglia (Frank et al., 2004, Mazzoni et al., 2007, Pekny et al., 2015). Prism adaptation impairments, including altered error reduction and reduced after-effects, have also been reported in these patients (Weiner et al., 1983, Stern et al., 1988, Canavan et al., 1990, Fernandez‐Ruiz et al., 2003). We propose that decision theory offers a unified conceptual framework within which to consider and identify possibly common computations (e.g. altered utility functions) that may explain both the classical cognitive impairments observed in Parkinson's disease (e.g. apathy) and concomitantly altered mechanisms of prism adaptation behaviour. Thus, re-casting accounts of prism adaptation behaviour within this computational framework offers a means of advancing conceptual and empirical understanding of cognitive impairments across different paradigms of enquiry.

How is the prediction *p(outcome*|*movement plan)* generated, and how is the predictive model (motor, proprioceptive, visual) adjusted during prism adaptation? The following two 4.3.2, 4.3.3 will offer a Bayesian answer to these questions.

#### 4.3.2 Bayesian statistics and optimal state estimation

**Fig. 5:**
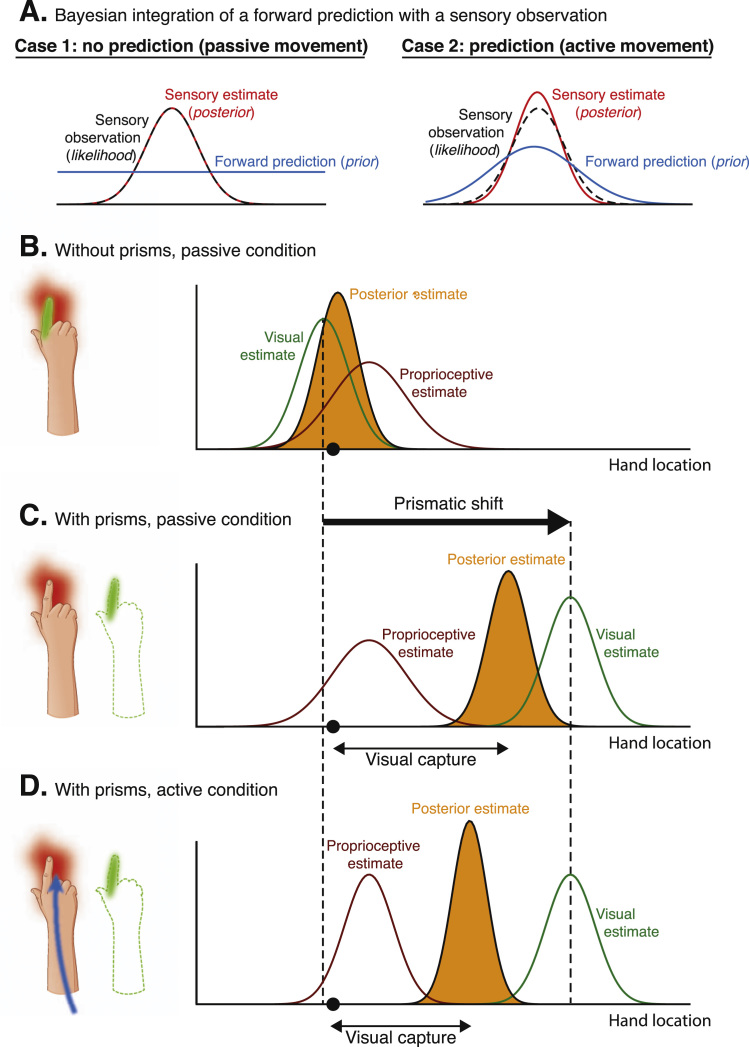
**Bayesian statistics in sensorimotor control.** Bayesian statistics describe how multiple sources of uncertain information can be combined optimally into a joint estimate. Here we consider the example of estimating the location of the hand in space. For all panels, the x-axis represents all possible locations of the hand and the y-axis is the probability of the hand being positioned at this location. **(A)** The optimal integration of a forward prediction (prior, in blue) with a sensory observation (likelihood, in black) is illustrated under two conditions. During a passive movement (case 1), no forward prediction is generated, i.e. the prior distribution is flat. The resulting sensory posterior estimate (in red) will therefore have the same distribution as the observation (likelihood). During an active movement however, a forward model generates a prediction of the next most likely hand position (prior) that can be integrated with sensory feedback (likelihood) to refine state estimation. This results in a posterior estimate (in red) that is more certain than the likelihood or the prior. **(B–D)** Bayesian statistics can also be used to describe (multimodal) visual-proprioceptive integration as illustrated here. The black dot on the x-axis represents the true location of the hand. **(B)** In the absence of prisms, the visual (green) and proprioceptive (red) estimates of the hand location are close to each other. Based on these two estimates, Bayes’ rule can be used to compute a posterior estimate of the hand location (purple) that is more certain than any of the sensory estimates. It posits that the relative level of uncertainty of the two sources of information (visual estimate and proprioceptive estimate) determines their relative contribution to the posterior estimate. Because the visual domain is more reliable (i.e. the width of the distribution is narrower), the resulting multimodal posterior estimate is biased towards the visual estimate. **(C)** The visuo-proprioceptive conflict induced by prism glasses is illustrated as a shift of the visual estimate (green) in the direction of the prismatic shift (towards the right). Bayes’ rule predicts that the resulting multimodal posterior estimate of the hand location is biased towards the visual observation because it is more reliable (i.e. lower standard deviation of the estimate) than the proprioceptive observation; this effect is called *visual capture*. The contribution of visual versus proprioceptive estimates of hand location to the multimodal posterior estimate is determined by their relative level of uncertainty. **(D)** In this condition, individuals actively move their limb without visual feedback prior to locating their hand in space. The execution of an active movement without visual feedback generates a proprioceptive forward prediction (case 2 of panel A) but no visual forward prediction (case 1 of panel A). If the proprioceptive forward prediction is accurate, the confidence in the resulting estimate will be increased. According to Bayes’ rule, the proprioceptive estimate will therefore have a greater influence on the multimodal posterior estimate of the hand location. In other words, the magnitude of visual capture is reduced. (For interpretation of the references to color in this figure legend, the reader is referred to the web version of this article.)

In order to generate appropriate motor commands given a movement plan, the nervous system needs to generate, update, and maintain accurate estimates of states of the body and the external world as we move through space (Franklin and Wolpert, 2011). During a pointing movement, multiple sources of error feedback (visual, proprioceptive, motor), with different time constants, both in-flight and following the reach endpoint, provide information about the hand and target position, and the discrepancy between the two. In addition, forward models generate advance predictions about expected outcomes before sensory observations occur. The nervous system must combine these multiple sources of information (predicted and observed) in order to generate a single integrated state estimate (e.g. position of the hand in space) that will guide behaviour (for review, see: Ernst and Bülthoff, 2004; Chandrasekaran, 2017). The uncertainty associated with any source of information places the problem of state estimation within a statistical framework. Bayesian statistics posit that inference about the state of the body or the external world can be made by combining and weighting each source of information according to its relative level of reliability. Consider for example the task of estimating the reach endpoint position of the hand after executing a pointing movement. Sensory predictions generated by forward models (e.g. proprioceptive forward model) constitute a prior assumption about where the hand should be if our internal models are correct. In Bayesian terms, such prior knowledge is represented as a *prior distribution* (blue curve in Fig. 5A). The sensory system also provides (noisy) information about the likely position of the hand (e.g. proprioceptive feedback). This information is represented as a *likelihood distribution* (dotted black curve in Fig. 5A). Combining both sources of information (prior and likelihood) according to Bayes’ rule offers a way to calculate a single, more certain estimate of the hand position, represented as a *posterior distribution* (red curve in Fig. 5A). Because it minimises uncertainty, such estimate is called *optimal*.

Bayes’ rule states that the probability of the hand being in a location *x,* given a proprioceptive observation *o*, is the product of the prior probability of the hand location (i.e. prediction about the most likely position of the hand) and the likelihood (i.e. visual estimate of the hand location), normalised by the probability of the observation. Mathematically, Bayes’ rule defines the posterior as follows:

(2) $$p\left(x|o\right)=\frac{p\left(x\right).p(o|x)}{p(o)}$$

Several studies have shown that, when integrating multiple sources of uncertain information, humans behave in a near optimal way that is well predicted by Bayesian models (Alais and Burr, 2004, Ernst and Banks, 2002, Kayser and Shams, 2015, Kording and Wolpert, 2004a, Körding et al., 2007, Sober and Sabes, 2005). Fig. 5B illustrates the use of Bayes’ rule for visual-proprioceptive integration under the assumption of normally distributed noise. The uncertainty (i.e. width of the Gaussian curve) associated with the proprioceptive estimate has been found to be greater than that of its visual homologue (Beers et al., 1996, van Beers et al., 1999, Ernst and Banks, 2002, Burge et al., 2010). Thus, according to Bayes’ rule, the optimal combination of proprioceptive and visual estimates of the hand location should result in a multimodal estimate that is biased towards the visual estimate.

In the context of prism adaptation, Bayesian statistics thus allow a re-formulation in mathematical terms of the perceptual phenomenon known as ‘visual capture’. Visual capture refers to the finding that individuals report their limb to be located closer to where it looks than where it feels under the visuo-proprioceptive conflict generated by prisms (Tastevin, 1937, Hay et al., 1965, van Beers et al., 1999). Traditionally, this phenomenon has been described qualitatively as “a kind of perceptual fusion of the discrepant visual and proprioceptive stimuli” where “the proprioceptive stimulus […] fail[s] to evoke its normal response” (Hay et al., 1965). By providing a rule to define optimal multisensory integration, Bayes’ theory makes quantitative predictions about the expected magnitude of sensory capture based on the level of uncertainty associated with information provided by each sensory modality (van Beers et al., 1999, Ernst and Banks, 2002, Burge et al., 2010) (Fig. 5BC). It also offers a theoretical explanation for the reduction in magnitude of visual capture that has been observed following an active hand movement compared to a passive one (Welch et al., 1979). The execution of a motor command elicits visual and proprioceptive forward models (efference copy) to predict the most likely next visual and proprioceptive observations (Lalazar and Vaadia, 2008). These predictions provide additional sources of information that can be combined with actual sensory observations to refine state estimation, i.e. reduce the uncertainty about the resulting percept. Because no visual feedback was provided during movement execution in Welch and colleagues’ experiment (Welch et al., 1979), only the proprioceptive estimate of the hand position could benefit from integration of the prediction generated by a (proprioceptive) forward model. We suggest that this explains why the resulting percept was more biased towards the proprioceptive estimate (i.e. lower visual capture) in this condition (Fig. 5D).

Visual capture has traditionally been studied relatively independently of prism adaptation, as it does not directly produce after-effects (Held and Hein, 1958, Welch et al., 1979). The next section, however, will illustrate how, when expressed in Bayesian terms, the phenomenon of ‘visual capture’ is an important factor that should influence the relative extent of proprioceptive versus visual internal model updating that occurs during prism adaptation.

#### 4.3.3 Internal model updating and credit assignment

Prism after-effects occur only when active but not passive movements are executed under prism exposure (Held and Hein, 1958, Welch et al., 1979). This is consistent with the idea that the generation of predictions prior to movement execution is fundamental to the ability to adapt in a way that generates after-effects. In a situation in which the reliability of sensory information is believed to be unchanged, persistent differences between predicted and measured observations implies the need for an update of internal models (Lalazar and Vaadia, 2008). The goal of this updating is to minimise systematic prediction errors and maintain internal consistency between predictions and observations.

In order to determine which internal model(s) to update (motor, visual, proprioceptive), the rate at which it should be updated, and for how long the update should be retained, the brain needs to solve what is known as a credit assignment problem, i.e. estimate the underlying *cause* of the prediction error, in order to assign learning to the correct internal model and update it appropriately (Wolpert et al., 2011). It has been proposed that the way in which credit is assigned can be inferred behaviourally by predicted effects on: the rate of error reduction, or the pattern of generalisation (to other contexts, spatial locations, body parts, modalities), or the timescale over which adaptation memory is retained (Cothros et al., 2006, Kording et al., 2007, Berniker and Kording, 2008, Kluzik et al., 2008, White and Diedrichsen, 2010).

When pointing towards visual targets while wearing prism glasses, there are many possible perturbations that could equally explain the occurrence of prediction errors. For example, a change in the arm dynamics – caused by fatigue for example – can alter the implementation of the motor command, causing systematic deviations from the predicted state. In this case, the nervous system should modify the relationship between a desired state and the associated motor command, i.e. adapt a *motor* internal model. Alternatively, prediction errors can arise from incorrect sensory estimates of the target location and/or of the position of the hand. In such a situation, the motor internal model does not necessarily need to be updated, but the *sensory* (visual and/or proprioceptive) internal models do.

**Fig. 6:**
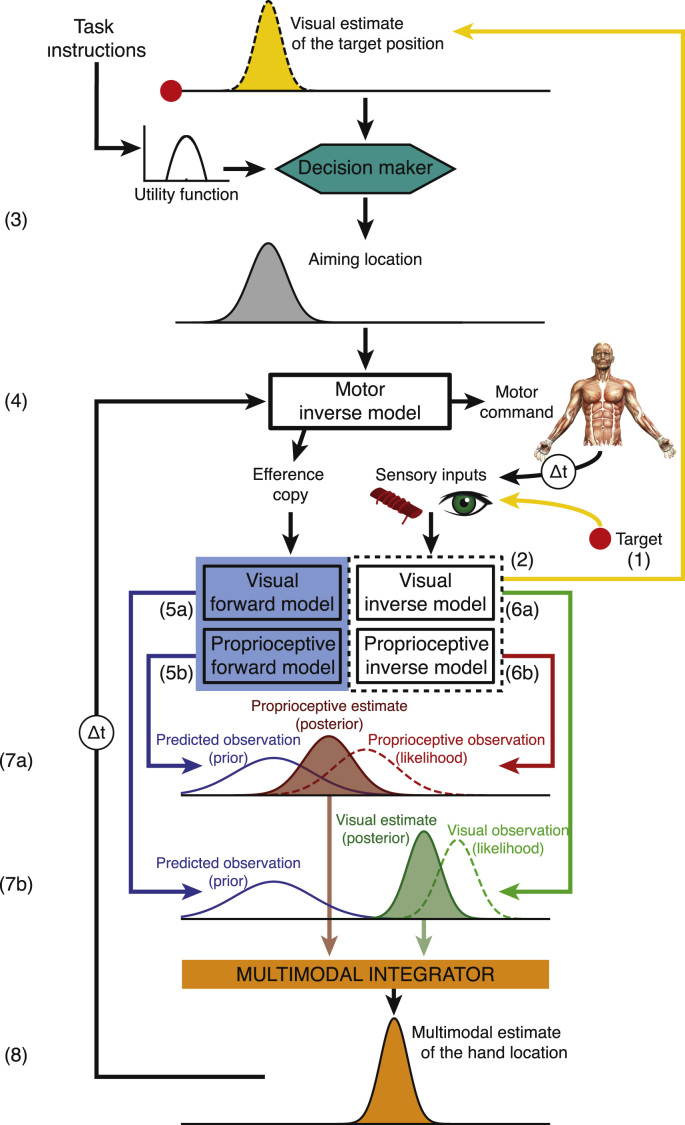
**A Bayesian account of prism adaptation.** This schema illustrates putative information processing occurring during the first reaching movement (closed-loop pointing) on trial 1 of prism exposure. First, light from the target is refracted through the prism lens and enters the eye inducing sensory input (yellow arrow between the target and the eye, 1). Based on this input, a visual inverse model generates an estimate of the most likely location of the target (yellow arrow between the visual inverse model and the visual estimate of the target location, top of Fig., 2). Owing to the prismatic displacement, this estimate will be right-shifted relative to the true location of the target in space (represented as the red dot, which is left of the visual estimate of the target location). The manual aiming direction believed to maximise expected utility, i.e. that which is expected to successfully align the reach endpoint with the target location, is selected by a decision maker (3). This process is explained in detail in Fig. 4. The selected aiming location is then fed to a motor inverse model, which transforms the desired goal (aiming direction) into an action plan to accomplish it (motor command, 4). An efference copy of this motor command is sent to visual (5a) and proprioceptive (5b) forward models that generate modality-specific predictions about the most likely next location of the hand. In this example, because a ballistic movement is generated (closed-loop pointing trial), this prediction concerns the hand position at the reach endpoint. Meanwhile, the execution of the motor command by the muscles generates visual and proprioceptive feedback (depicted by the arrows originating from the muscle spindle and eye symbols) that is integrated by sensory inverse models to generate modality-specific estimates of the sensed location of the hand (6a: visual and 6b: proprioceptive estimate of the hand location at the reach endpoint). Bayesian integration of the modality-specific forward predictions (prior) with the sensory observations (likelihood) generates modality-specific estimates of the most likely hand location (posterior) (7a and 7b). See Fig. 5A for a detailed description of this type of integration. The resulting visual and proprioceptive (posterior) estimates are then combined into a multimodal (visuo-proprioceptive) posterior estimate of the hand position (8). See Fig. 5B–D for a detailed description of this type of integration. On trial 1 of prism exposure, the rightward prism displacement induces: 1) leftward performance error (i.e. divergence between the visual estimate of the target position, 2, and the multimodal estimate of the hand location after movement execution, 8); 2) prediction errors in the sensory internal models (i.e. divergence between prior and likelihood, 7a–b); 3) visuo-proprioceptive conflict (i.e. divergence between the proprioceptive and visual estimates of the hand location, 8). The effect of internal model updating is to reduce these performance errors and to gradually realign all the distributions depicted in this schema (i.e. aiming location, predicted visual and proprioceptive observations, visual and proprioceptive observations, visual and proprioceptive estimates, multimodal estimate). Internal models are updated iteratively within this network as evidence accumulates and performance is adjusted from one trial to the next. In order to update internal models appropriately, the brain has to attribute learning to the correct internal models (motor, visual or proprioceptive). The level of uncertainty associated with each source of information (sensory predictions, sensory observations, sensory estimates) at every level of integration (modality-specific and multimodal) is used to infer the most likely error source and hence assign learning to the correct internal model. In this example, the visual estimate is more certain than the proprioceptive estimate, which should result in a preferential update of proprioceptive internal models. This model architecture incorporates the ‘strategic control versus spatial realignment’ dissociation as performance error can alternatively be quickly corrected at the level of the decision maker by selecting an aiming location that would result in a negative disparity between the visual estimate of the target position and the multimodal estimate of the reach endpoint hand location (see Fig. 3 for a detailed description). (For interpretation of the references to color in this figure legend, the reader is referred to the web version of this article.)

Credit assignment refers to the complex problem of attributing an error to its causal source (Kluzik et al., 2008). It requires the nervous system to take into account a large number of parameters, which could include contextual cues, volatility of the environment, and uncertainty in the estimates, predictions, and sensory observations. Computational models incorporating a Bayesian estimator of the source of the error signal have been able to successfully explain post-adaptation generalisation (Berniker and Kording, 2008, Haith et al., 2009). For example, using a Bayesian estimator of the visual, proprioceptive and motor contributions to the overall perturbation, Haith et al. (2009) generated and confirmed the unintuitive prediction that even a purely motor disturbance, involving no intersensory conflict (force-field), would lead to sensory adaptation, because of uncertainty in the source estimates. Thus, to determine which internal model to update (visual, proprioceptive, or motor), it appears crucial to have a computational architecture in which the uncertainty associated with each source of information is represented (Berniker and Kording, 2011). Fig. 6 illustrates such architecture. Under most normal daily circumstances (e.g. not wearing prisms), the uncertainty associated with the predicted landing position of the finger (output of level 3 in Fig. 6) makes the motor system the most likely source of the error, which should cause preferential updating of the motor inverse model (Kawato, 1999, Kording et al., 2007, Inoue et al., 2016). If credit assignment proceeds in a statistical fashion, a non-zero probability that the error arose from faulty sensory internal models should be inferred. In order to determine which of the visual or proprioceptive internal models is more likely to be responsible for the prediction error, the modality-specific and cross-modal levels of integration (levels 7 and 8 in Fig. 6 respectively) should be considered. At the modality-specific levels of integration (i.e. visual prior/likelihood, proprioceptive prior/likelihood, see levels 7a and 7b of Fig. 6), information theory offers a way to determine whether the prediction error (i.e. divergence between prior and likelihood) should be attributed to faulty forward or inverse models. Low confidence in the prediction (i.e. uncertain prior) and/or high confidence in the sensory observation (i.e. certain likelihood) imply the need for a sensory forward model update. Alternatively, high confidence in the prediction (i.e. certain prior) and/or low confidence in the sensory observation (i.e. uncertain likelihood) indicate the need for a sensory inverse model update. Degrading the quality of the visual feedback of the reach endpoint error (e.g. by blurring the target or by reducing the duration of the terminal visual feedback) is a way to experimentally manipulate the level of reliability of the visual observations, thus potentially maximising sensory inverse model updating. At the cross-modal level of integration (level 8 of Fig. 6)**,** comparison of the uncertainty in the visual versus proprioceptive estimates should guide the neural system towards an update of the visual proprioceptive model. In the case of a visuo-proprioceptive conflict (i.e. divergence between the visual and proprioceptive estimates), the sensory internal models associated with the most uncertainty (visual or proprioceptive) are the ones that should be preferentially updated. Because this computational architecture (Fig. 6) incorporates the level of uncertainty associated with each source of information (width of the Gaussian curve) at every level of integration, there is sufficient information to determine which internal model (visual, proprioceptive and/or motor) should be updated, and how it should be updated.

Compared to the traditional dual-process model, this framework makes quantitative predictions and offers a principled explanation of the predicted behavioural consequences of modulating the uncertainty of specific information sources (for example, see: Kording and Wolpert, 2004a; Burge et al., 2010; Yamamoto and Ando, 2012). For example, this can explain why terminal prism exposure (no visual feedback during reaching movements, endpoint error feedback only) induces greater visual than proprioceptive after-effects, while concurrent prism exposure (visual feedback during reach movements and endpoint error) induces greater proprioceptive than visual after-effects (Redding and Wallace, 1996, Redding and Wallace, 2001, Redding and Wallace, 2006b). The occlusion of visual feedback under terminal prism exposure conditions increases the uncertainty of the visual estimate of the hand position. Hence, when the prisms induce a prediction error, the cause of this is more likely to be attributed to a faulty visual internal model, thus resulting in greater visual than proprioceptive model updating. By contrast, the presence of visual feedback in concurrent prism exposure conditions allows for continuous state estimation to occur in both the visual and proprioceptive domains. It is known in such condition that visual estimates are more certain relative to proprioceptive estimates (Ernst and Banks, 2002, also see section 4.2.1 on visual capture and Fig. 4; Burge et al., 2010). Hence, when a prediction error is experienced at the reach endpoint (under concurrent prism exposure), it is more likely to be attributed to a faulty proprioceptive internal model, which is then updated accordingly. Based on this interpretative framework, we predict that, across individuals, the magnitude of visual capture (which quantifies relative confidence in visual versus proprioceptive information) should predict the degree to which individuals update their visual relative to their proprioceptive internal models under concurrent prism exposure (i.e. the relative difference in after-effect magnitude between visual straight ahead judgement and proprioceptive straight ahead pointing).

## 5 Conclusion

Prism adaptation is one of the oldest experimental paradigms used in the sensorimotor adaptation literature (Von Helmholtz, 1867). Yet, it has been studied mostly within a traditional cognitive psychology theoretical framework, which has important explanatory limitations. In this paper, we have advocated for the utility of a computational framework that re-conceptualises prism adaptation in terms of its constituent algorithms. In our view, the advantages of a computational approach are several. First, Bayesian decision theory allows a precise quantitative re-formulation of 'strategic control' and 'spatial realignment' concepts, within the same utility framework used in other task contexts, such as reward-guided learning and decision-making (Frank et al., 2004, Daw et al., 2006, Behrens et al., 2007). This shared conceptual and mathematical language opens up the possibility to investigate experimentally potential commonalities in the functional and neural mechanisms engaged across these very different task contexts. For example, one could hypothesise that there are some shared neural substrates responsible for aspects of the computation of expected utility notwithstanding the different types of action outcome experienced in these different classes of task. Second, Bayesian statistics provides a mathematical framework that specifies how spatial realignment should proceed, and thus offers quantitative explanation crucially lacking in the traditional framework. Third, state-space models offer a simple mathematical description of the temporal dynamics of internal estimates thought to underlie prism adaptation behaviour, moving beyond the categorical dualist approach of the traditional framework. This approach enables quantitative questions about information processing and neural implementation, such as: 1) how many processes best explain prism adaptation behaviour?, 2) is there a discrete number or a continuum of processes/timescales which varies with task parameters?, or 3) are distinct brain regions associated with distinct timescales, or might a given brain circuit have the ability to perform similar computations over a range of differing timescales?

To conclude, we submit that progress in understanding the functional and neural bases of prism adaptation behaviour requires this experimental paradigm to be re-conceptualised at an algorithmic level of description. Doing so offers our field the opportunity to capitalise on explanatory gains generated by the literature on computational sensorimotor control. Such insights are being leveraged in the literature on other kinds of adaptation task, but the prism literature has so far remained oddly immune. Given the distinctive features of prism adaptation, and its applications in neuropsychology, we believe the time for our field to start leveraging these gains is ripe.
